# Supplementary material for: Pore-forming Esx proteins mediate toxin secretion by Mycobacterium tuberculosis
Source: Nat Commun. 2021 Jan 15;12:394. doi: 10.1038/s41467-020-20533-1 (PMC7810871; doi:10.1038/s41467-020-20533-1)
Supplement: Supplementary file 1 — Supplementary Information [file 41467_2020_20533_MOESM1_ESM.pdf]

SUPPLEMENTARY INFORMATION

Pore-forming Esx proteins mediate toxin secretion by *Mycobacterium tuberculosis*

Uday Tak, Terje Dokland, and Michael Niederweis

Department of Microbiology, University of Alabama at Birmingham, 845 19th Street South, Birmingham, AL 35294, USA

Running title: Toxin secretion by *Mycobacterium tuberculosis*

*Address correspondence to: Michael Niederweis, mnieder@uab.edu

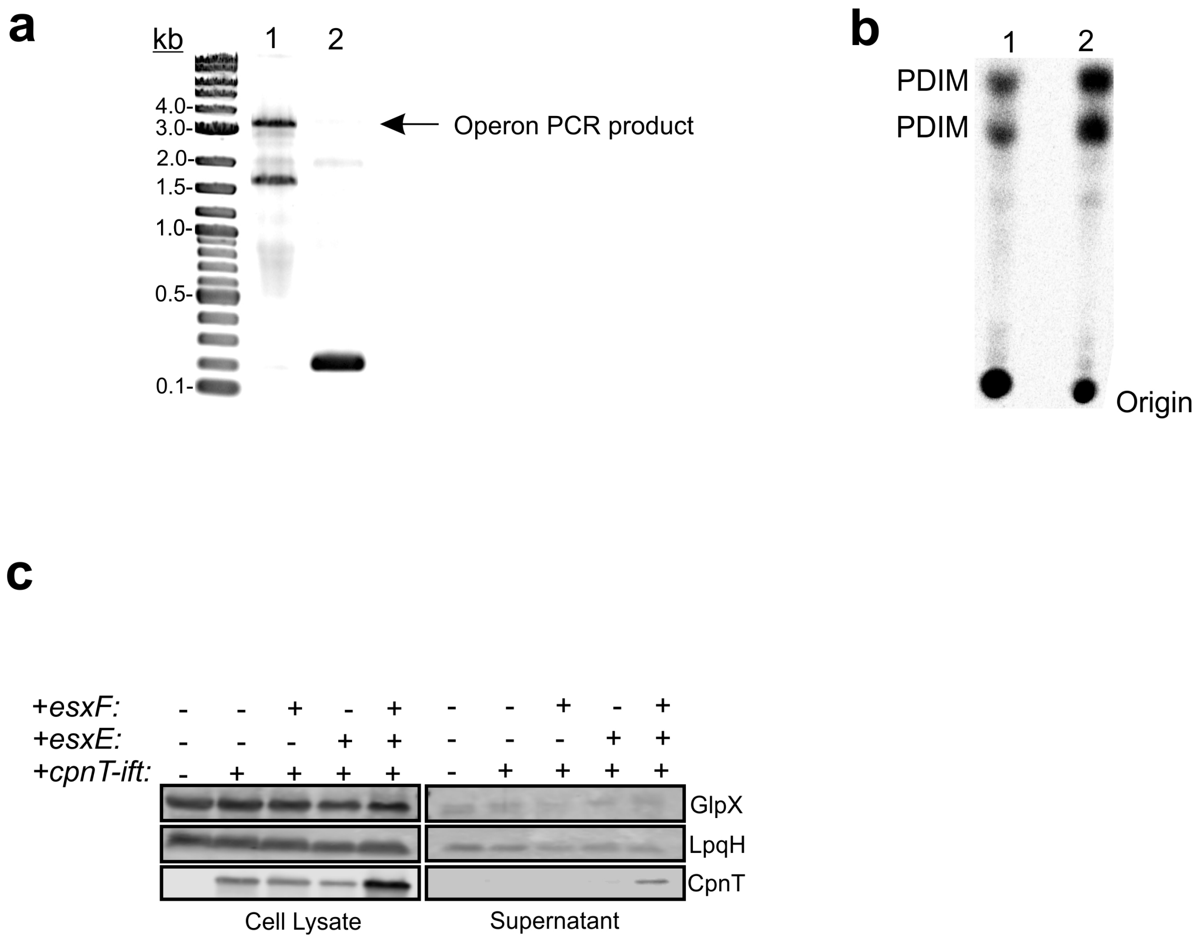

Figure S1. Characterization of the *cpnT* operon mutant of *M. tuberculosis.*

a. **Analysis of the *cpnT* operon deletion of Mtb.** PCR analysis of the Mtb mc^{2}6206 Δ*esxFE-cpnT-ift* (ML2016) and of the parent strain Mtb mc^{2}6206 used in this study (Table S2). Genomic DNA was amplified with EsxF-FWD-NdeI (forward primer) and IFT-REV-HindIII (reverse primer) for an expected product size of 3.6 kb (Table S4) Lane 1 indicates PCR amplification of wild type strain, and lane 2 indicates the Δ*esxFE-cpnT-ift* strain. Experiment was performed once.
b. **Analysis of pthiocerol dimycocerosates (PDIM) in the *cpnT* operon mutant of Mtb*.*** Thin layer chromatography of lipid fraction from the indicated strains labelled with ^{14}C-propionate confirming the presence of the outer membrane lipid pthiocerol dimycocerosates (PDIM). Lane 1 indicates the wild type strain and lane 2 indicates the Δ*esxFE-cpnT-ift* strain. Experiment was performed once.
c. **Secretion of CpnT *in vitro.*** The indicated Mtb strains were grown in AMTB medium without tyloxapol. The supernatant was filtered and concentrated. secretion assay for detection of CpnT in the culture supernatant using the indicated strains. Representative of at least 3 experiments.

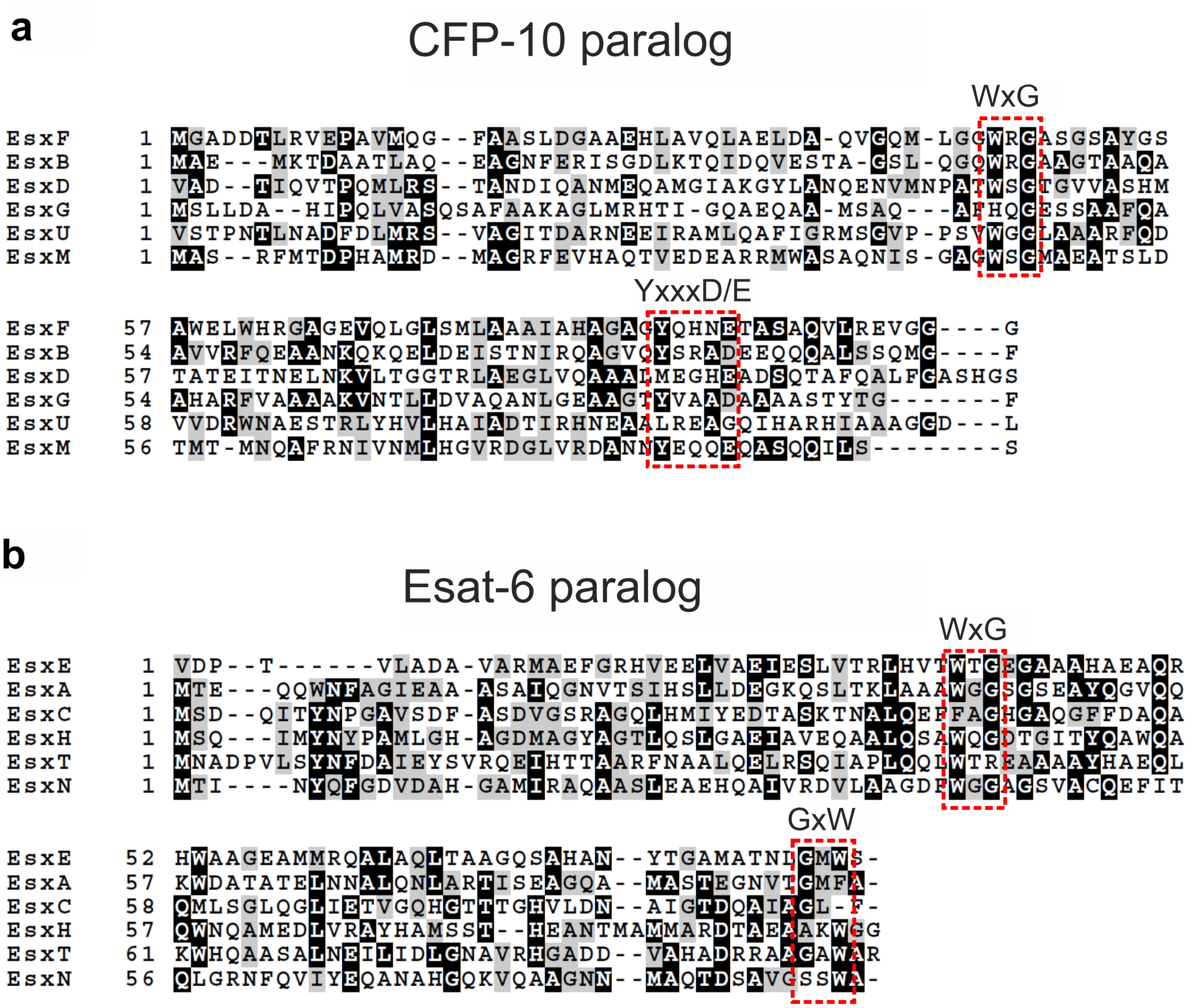

Figure S2. Sequence alignment of ESAT-6 and CFP-10 paralogs
Multiple sequence alignment of (**A**) CFP-10 family proteins (EsxF paralogs) and (**B**) ESAT-6 family proteins (EsxE paralogs). The locations of the WXG and the YXXXD/E motif is indicated in the dashed red boxes. Also indicated is a conserved reverse WXG (GXW) motif found on the c-terminus of ESAT-6 homologues. Sequences were obtained from Mycobrowser and aligned in T-Coffee Expresso. The alignment was visualized using the BoxShade server.

a

EsxF

1	MGADDTLRVEPAVMQGFAASLDGAAEHLAVQLAELDAQVGQMLGGWRGAS (WxG)	50
51	GSAYGSAWELW**HRGAGEVQLGLSMLAA**AIAHAGAGYQHNETASAQVLREV	100
101	GGG (YxxxE)	103

EsxE

1	VDPTVLADAVARMAEFGRHVEELVAEIESLVTRLHVTWTGEGAAAHAEAQ (WxG)	50
51	RHWAAGEAMMRQALAQLTAAG**QSAHANYTGAMATNLG**MWS	90

Extracellular

Membrane Interaction / Transmembrane Helix

AxxxA / GxxxG α-helical interaction motif

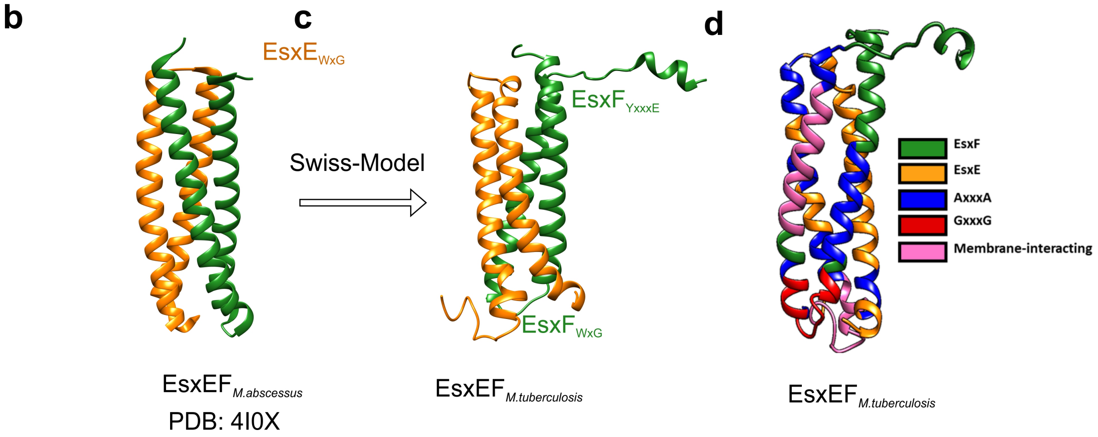

Figure S3. *In-silico* analysis of the EsxE-EsxF heterodimer

a. MemSAT analysis of predicted structural features in EsxF and EsxE. Memsat was used to calculate predicted structural features with extracellular and membrane-interacting / transmembrane segments indicated. AXXXA and GXXXG motifs were also identified and indicated in teal colors.

b. Structure of EsxE-EsxF homolog from *Mycobacterium abscessus* (PDB 4I0X), hereafter referred to as $EsxEF_{Mabs}$.

c. Swiss model generated structure using $EsxEF_{Mabs}$ and EsxAB as a homology template. Hereafter referred to as $EsxEF_{Mtb}$. The locations of the T7SS motifs are indicated: EsxF-WXG (W1), EsxE-WXG (W2), and EsxF-YXXXE.

d. EsxE-EsxF model with the indicated features highlighted.

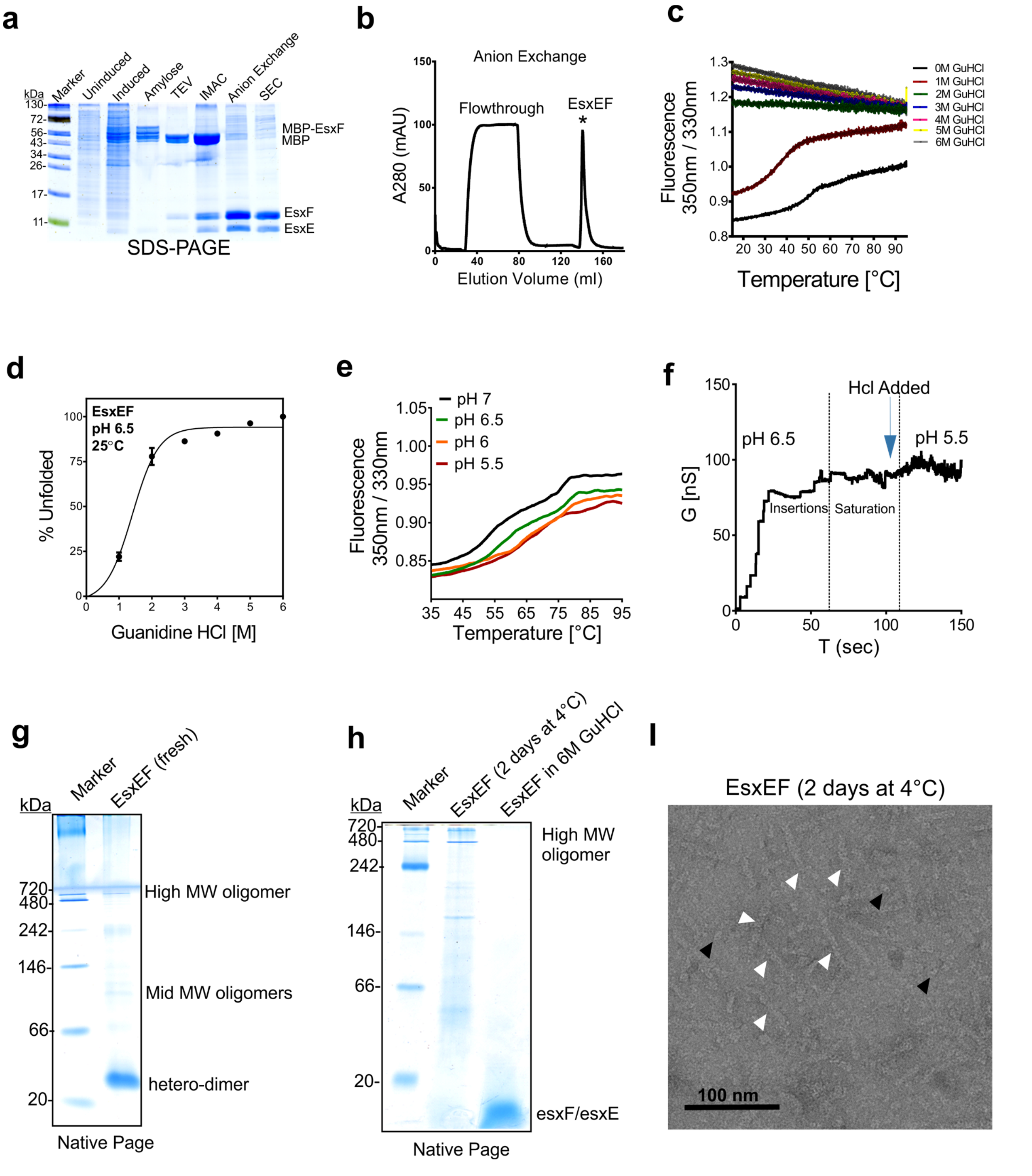

Figure S4. Purification and biochemical analysis of the recombinant EsxE-EsxF complex.

a. Colloidal Coomassie-stained SDS PAGE gel indicating the purification steps and subsequent purity of EsxEF produced from *E. coli*. Representative of at least 3 purifications.
b. Anion exchange chromatogram of the "IMAC" fraction from panel A run on a HiTrap QFF anion-exchange column using a Bio-Rad Duoflow HPLC system. Flow-through indicates the residual MBP which elutes at 150 mM NaCl. The sharp peak indicated with an asterisk (*) indicates pure EsxEF. Further purification details can be found in methods.

c. Nano-differential scanning fluorometry of EsxEF in 0 – 6 M guanidine hydrochloride to determine global folding of the protein. Data represents tryptophan fluorescence at 350nm (exposed to solvent) vs 330nm (buried in protein) as a function of the temperature. Data was collected on a NanoTemper Promethus at the UAB structural biology core facility.
d. Percent unfolding of EsxEF at 25°C for which all experiments were conducted derived from panel C data. The values for 6 M Guanidine hydrochloride were set to 100% and the values for 0 M were set to 0%. A sigmoidal fit was performed using Graphpad.
e. EsxEF tryptophan fluorescence measurements performed on a NanoTemper Tycho in 25 mM sodium phosphate 150 mM NaCl at the indicated pH.
f. Test of EsxEF channel gating by pH. Representative current trace indicating EsxEF insertions in DphpC membranes at pH 6.5 (labelled "Insertions)" which were then allowed to continue until saturation (labelled "saturation"). HCl was then added to the bilayer cuvette until the pH dropped to 5.5 (blue arrow "HCl added") and data was recorded further. Representative of 5 membranes.
g. Native polyacrylamide gel indicating freshly purified EsxEF. Indicated are the predicted heterodimer and oligomers of increasing sizes. Representative of at least 2 experiments.
h. Native polyacrylamide gel of EsxEF left at 4°C for two days after purification and in 6 M guanidine hydrochloride (GuHCl). Representative of at least 2 experiments.
i. Electron micrograph of EsxEF (2 days at 4°C) stained with 1% uranyl formate showing the conversion of EsxEF to filaments (white triangles). Black triangles indicate low molecular weight EsxEF pores. Data is from a single experiment on the sample which was left at 4°C for 2 days.

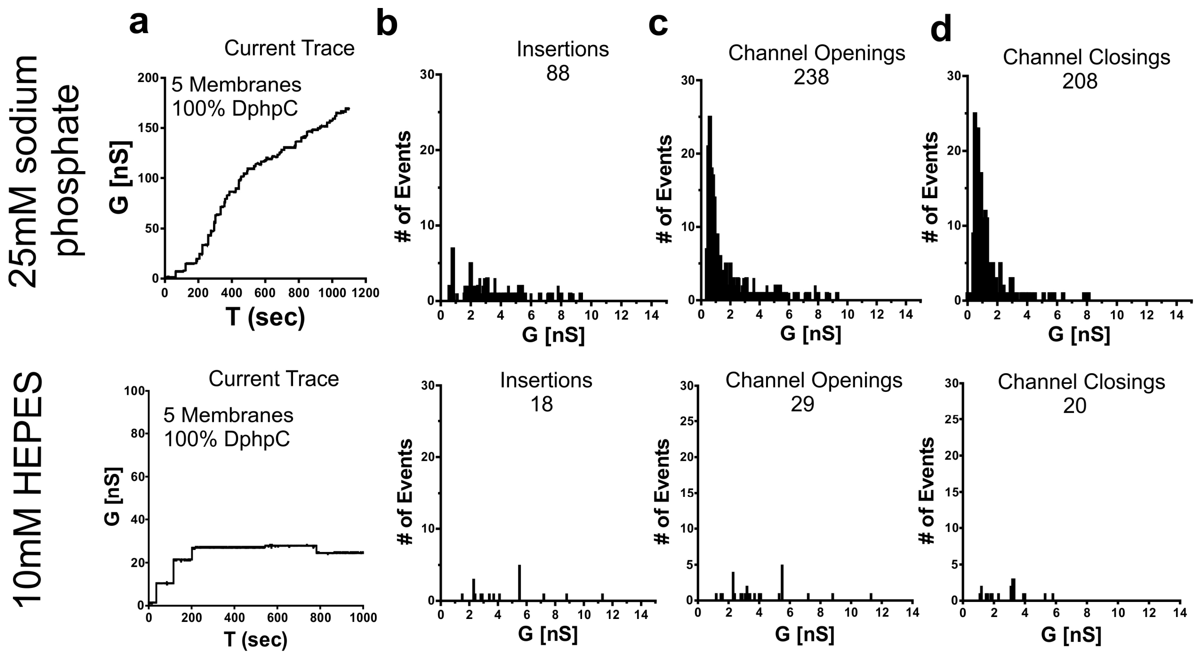

Figure S5. Phosphate dependent pore-formation by EsxE-EsxF complex

a. Current Trace and total number of channel-insertion events in 25 mM sodium phosphate 1 M KCl pH 6.5 (Top) or 10 mM HEPES, 1M KCl pH 6.5 (bottom panel).
b. Conductance values recorded for all insertion events in phosphate (top) or HEPES (bottom) buffer.
c. Channel opening events in phosphate (top) or HEPES (bottom) buffer.
d. Channel closing events in phosphate (top) or HEPES (bottom) buffer.

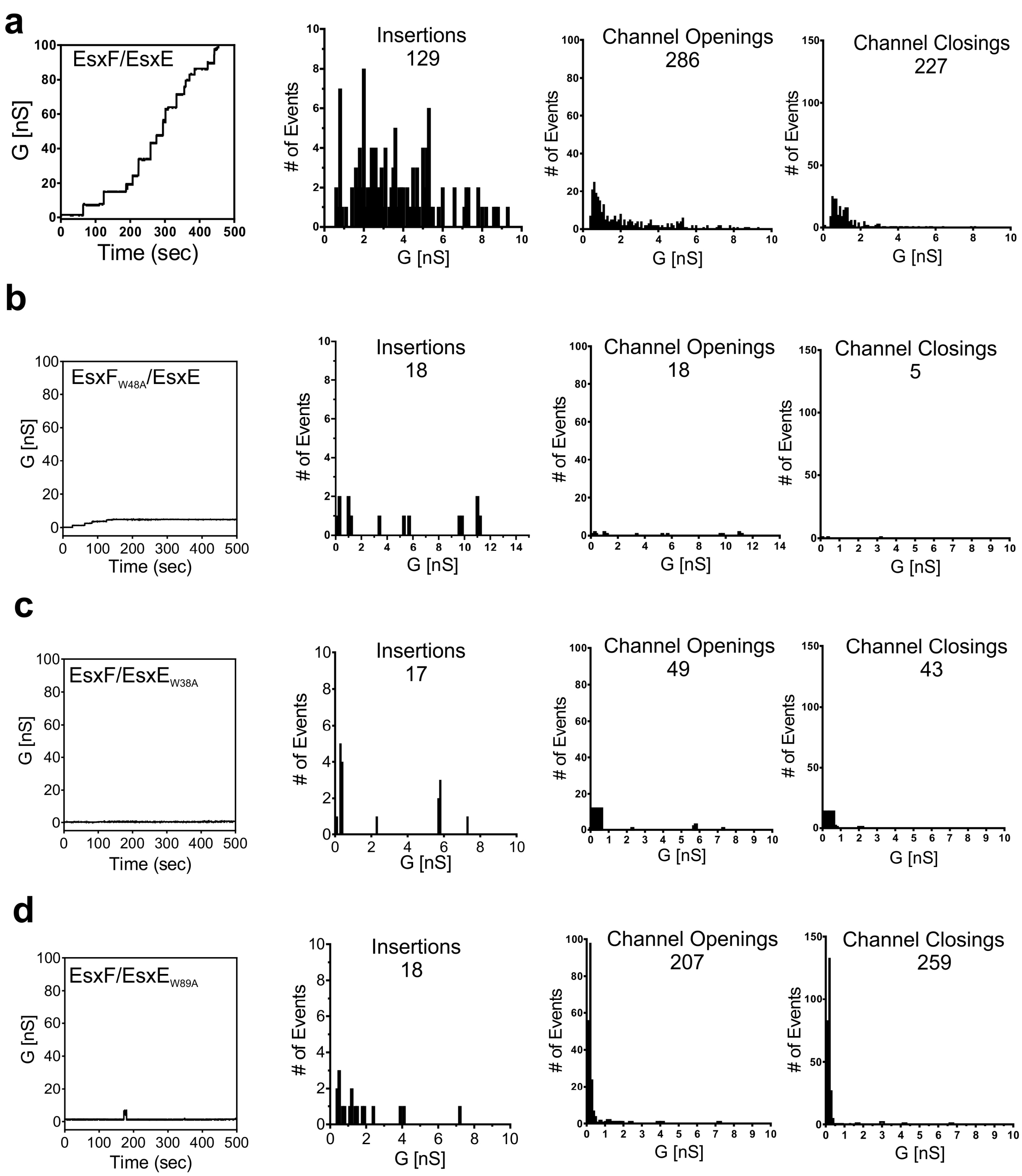

Figure S6. Channel activity of the wt and mutated EsxE-EsxF complexes by lipid bilayer experiments.

The planar lipid bilayer experiments were done in 25 mM sodium phosphate 1 M KCl, at -10 mV in diphytanoyl phosphatidylcholine (DPhPC) membranes for EsxEF (a) and the indicated WxG variants (b-d). From left to right per row: representative current trace, total channel insertion events, total channel opening events, and total channel closing events. A total of 10 membranes were performed using 1 µg of protein added to the cis and trans side. This figure accompanies figures 4 and 5.

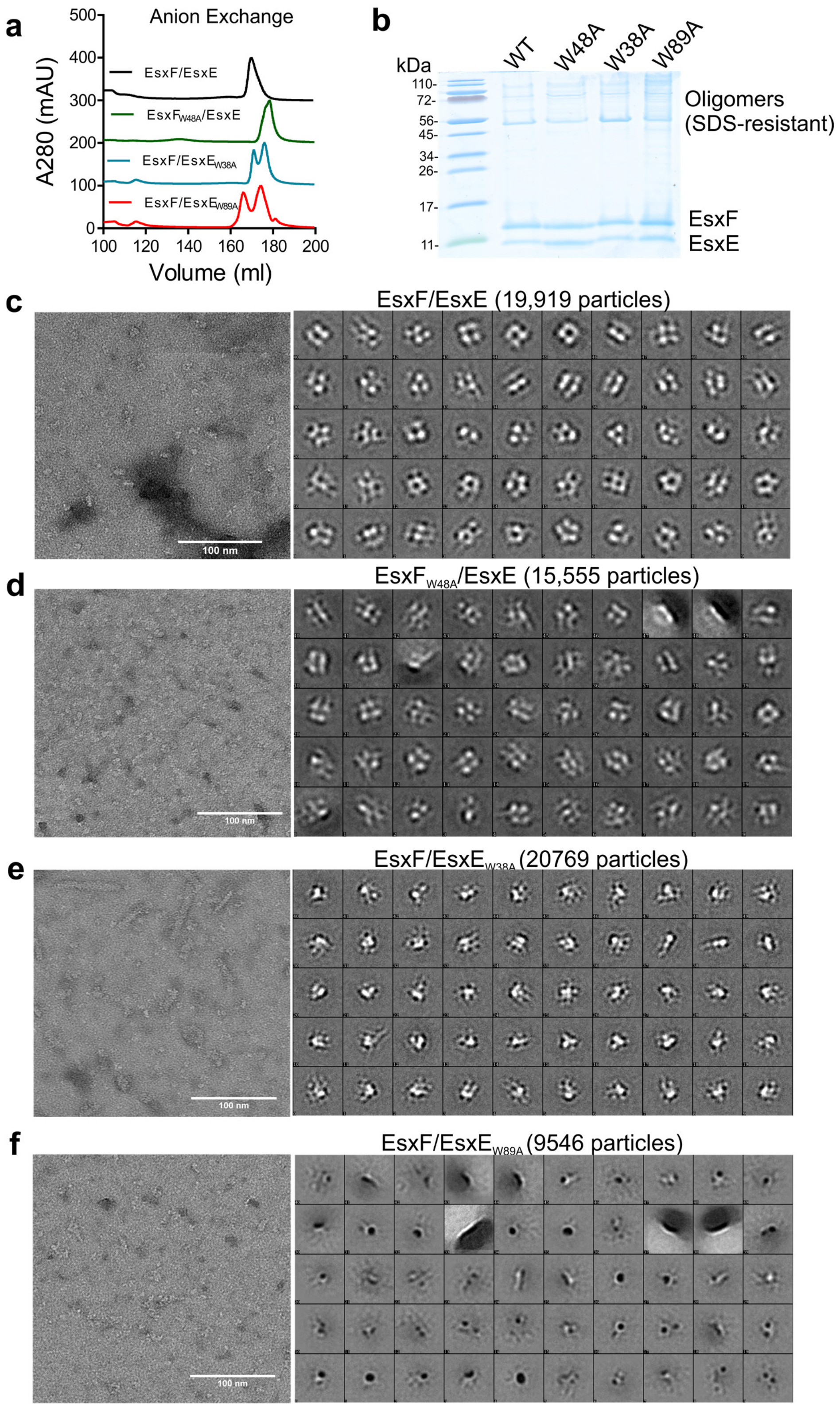
a
Anion Exchange
A280 (mAU)
500
400
300
200
100
0
100
120
140
160
180
200
Volume (ml)
EsxF/EsxE
EsxFW48A/EsxE
EsxF/EsxEW38A
EsxF/EsxEW89A
b
kDa
WT
W48A
W38A
W89A
110-
72-
56-
45-
34-
26-
17-
11-
Oligomers
(SDS-resistant)
EsxF
EsxE
c
EsxF/EsxE (19,919 particles)
100 nm
d
EsxFW48A/EsxE (15,555 particles)
100 nm
e
EsxF/EsxEW38A (20769 particles)
100 nm
f
EsxF/EsxEW89A (9546 particles)
100 nm

Figure S7. Purification and electron microscopy of WXG mutants of the EsxE-EsxF complex.

a. Anion exchange chromatograms of the indicated EsxEF WxG variants compared to wild type.

b. Coomassie-stained SDS-PAGE gel of the anion exchange fractions indicating the presence of EsxF and EsxE proteins and SDS-resistant oligomers. Representative of at least two purifications.

c. Raw electron micrograph of 50 nM EsxEF (C) or the indicated WxG variant: $EsxF_{W48A}$/EsxE (**d**), EsxF/$EsxE_{W38A}$ (**e**) or EsxF/$EsxE_{W89A}$ (**f**) stained with 1% uranyl formate. Shown are the full reference-free 2D-class averages for each protein calculated in EMAN2.2. 2D averages were collected from a single EM session. At least two separate protein preparations were screened by EM for WT, $EsxF_{W48A}$/EsxE, EsxF/$EsxE_{W38A}$, and one for EsxF/$EsxE_{W89A}$.

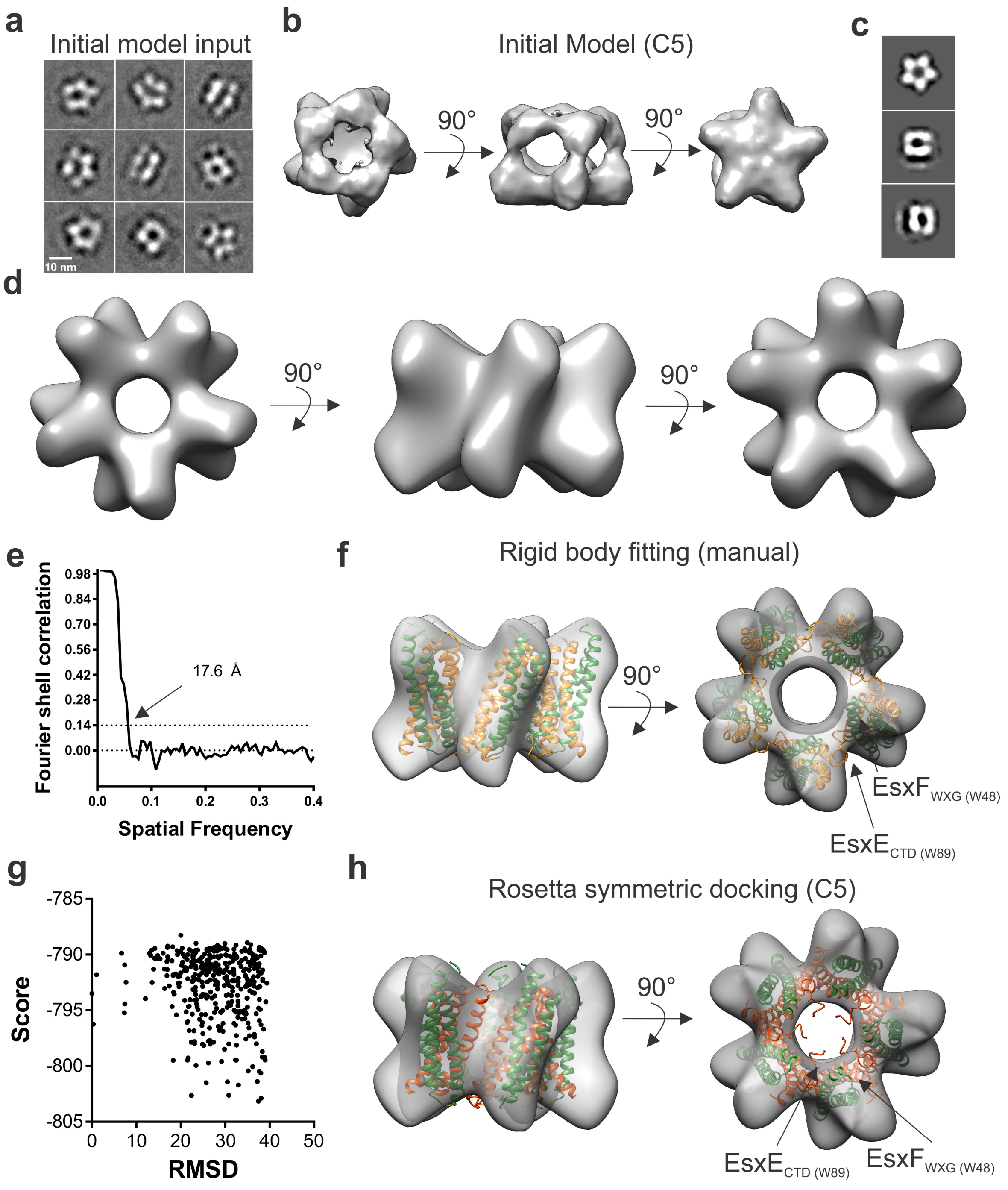

Figure S8. Negative stain reconstruction of EsxEF.

a. Reference-free 2D class averages used for initial model generation. Data are from a single EM session.

b. Initial model generated with C5 symmetry from input averages shown in A.

c. Projection images from initial model shown in B. Data are from a single EM session.

d. 3D refinement of initial model with C5 symmetry.

- **e.** Fourier shell correlation of the refined 3D density map shown in D.
- **f.** Rigid body fitting of five copies of the EsxF/EsxE homology model in chimera. The disordered CTD of EsxF was truncated for fitting. Indicated are the locations of the EsxF WXG motif and the EsxE CTD (GXW) motif.
- **g.** Score vs root mean squared deviation plot from 1000 structures generated by the Rosetta symmetric docking program using C5 symmetry and the EsxF/EsxE homology model as an input.
- **h.** Lowest free energy *de-novo* Rosetta structure of EsxF/EsxE fit as a rigid body into the negative stain density map using Chimera.

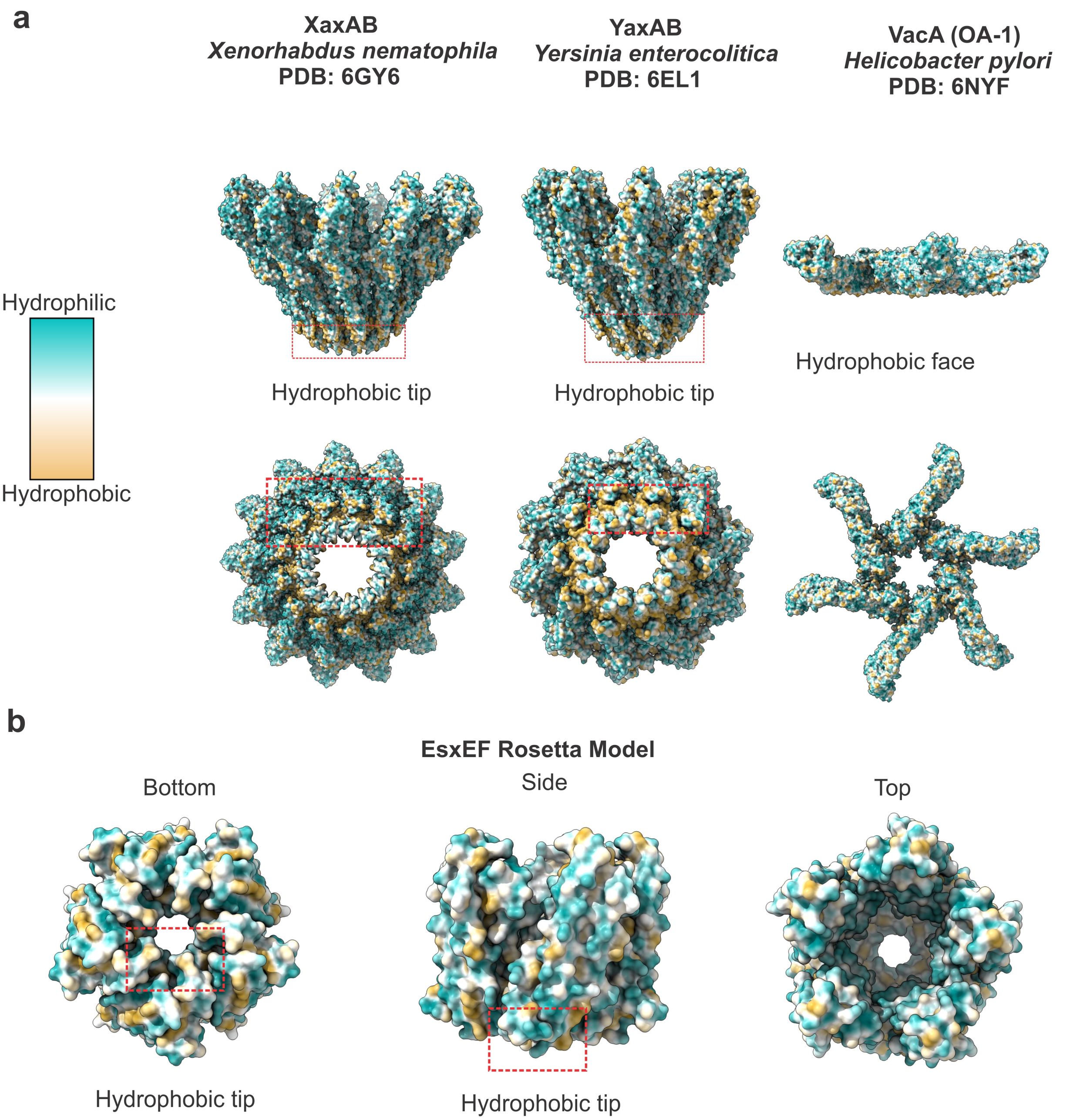

Figure S9. Comparison of EsxEF model with pore-forming proteins.

a. Surface structures of the indicated pore-forming toxins colored by hydrophobicity using ChimeraX. The side and bottom views are represented and the location of the hydrophobic tip and / or hydrophobic face of the oligomer is indicated by red dashed lines. PDB 6GYG (Schubert et al, *eLife* 2018), PDB 6EL1 (Brauning et al, *Nat. Communications* 2018), PDB 6NYF (Zhang et al, *PNAS* 2019.

b. EsxEF Rosetta model (described in Figure S8) represented by hydrophobicity. The location of a putative hydrophobic tip formed by tryptophan 89 of EsxE is indicated in red dashes. Bottom, side, and top assignments are hypothetical based on the model.

Table S1. Plasmids used in this study

Plasmid	Properties	Reference
pML3924	*E. coli* expression vector: pBR322 origin, *bla, lacI*^{q} , P$_{T7lac}$ His6-*malE*-polyN-TEV-*esxF-esxE*$_{WT}$	This study
pML3938	mycobacterial expression vector: episomal, hyg^{R} ,pAL5000: psmyc-*cpnT-ifT*	This study
pML3941	mycobacterial expression vector: integrative, kan^{R} ,attL5: psmyc-*esxF-esxE*	This study
pML3954	mycobacterial expression vector: integrative, kan^{R} , attL5: psmyc-*esxF*$_{W48A}$-*esxE*	This study
pML3955	mycobacterial expression vector: integrative, kan^{R} , attL5: psmyc-*esxF*$_{Y86A}$*F-esxE*	This study
pML3956	mycobacterial expression vector: integrative, kan^{R} ,attL5: psmyc-*esxF*	This study
pML3957	mycobacterial expression vector: integrative, kan^{R} ,attL5: psmyc-*esxE*	This study
pML3958	mycobacterial expression vector: integrative, kan^{R} ,attL5: psmyc-*esxF-esxE*$_{W38A}$	This study
pML3962	*E. coli* expression vector vector: pBR322 origin, *bla, lacI*^{q} , P$_{T7lac}$ His6-*malE*-polyN-TEV-*esxF*$_{W48A}$-*esxE*	This study
pML3963	*E. coli* expression vector vector: pBR322 origin, *bla, lacI*^{q} , P$_{T7lac}$ His6-*malE*-polyN-TEV-*esxF-esxE*$_{W38A}$	This study
pML4512	*E. coli* expression vector vector: pBR322 origin, *bla, lacI*^{q} , P$_{T7lac}$ His6-*malE*-polyN-TEV-*esxF-esxE*$_{W89A}$	This study
pML4514	mycobacterial expression vector: integrative, kan^{R} , attL5: psmyc-*esxF-esxE*$_{W89A}$	This study
pML4515	mycobacterial expression vector: integrative, kan^{R} , attL5: psmyc-*esxF-esxEΔAA87-90*	This study

Table S2. Mycobacterial strains used in this study

Strain	Source	Reference / Identifier
M. tuberculosis mc^{2}6206 H37Rv derivative: Δ*leuCD,* Δ*panCD.* Auxotroph BSL-2 strain. Parent strain.	Dr. William R Jacobs Jr. (Albert Einstein University)	(Sampson et al., 2004)
M. tuberculosis mc^{2}6206 Δ*esxF-esxE-cpnT-ifT*	This study	ML2016
ML2016 / pML3938	This study	ML2625
ML2016 *attL5*::pML3941 / pML3938	This study	ML2635
ML2016 *attL5*::pML3954 / pML3938	This study	ML2636
ML2016 *attL5*::pML3955 / pML3938	This study	ML2637
ML2016 *attL5*::pML3956 / pML3938	This study	ML2638
ML2016 *attL5*::pML3957 / pML3938	This study	ML2639
ML2016 *attL5*::pML3958 / pML3938	This study	ML2640
ML2016 *attL5*::pML4514 / pML3938	This study	ML2684
ML2016 *attL5*::pML4515 / pML3938	This study	ML2685

Table S3. Antibodies used in this study

Antibody	Source	Identifier
Rabbit polyclonal anti-TNT	Genscript	(Danilchanka et al., 2014)
Rabbit polyclonal anti-EsxF	Genscript	This study.
Rabbit polyclonal anti-EsxE	Genscript	This study.
Rabbit polyclonal anti-IFT	Genscript	(Sun et al. 2015)
Rabbit polyclonal anti-GlpX	UAB Hybridoma core	(Speer et al., 2015)
Mouse monoclonal anti-LpqH	BEI Resources	NR-13792
Mouse monoclonal anti-RNA Polymerase β Subunit	Biolegend	Cat # 663903
Mouse monoclonal anti-MctB	UAB Hybridoma core	(Mitra et al., 2017; Speer et al., 2015)
Donkey anti-rabbit IgG (H+L)-Alexafluor-594 secondary	Thermo Fisher	Cat # A-21207
Goat anti-rabbit IgG-FITC secondary	Sigma Aldrich	12-507
IRDye 680RD Donkey anti-rabbit IgG secondary	LI-COR	926-68073
IRDye 800CW Goat anti-mouse IgG Secondary	LI-COR	926-332210

Table S4. Oligonucleotides used in this study

Oligonucleotide name	Sequence
EsxF-NdeI-FWD	AGCCATATGGGTGCCGACGACACGCT
EsxE-HindIII-Rev	GGCCGCAAGCTTTCACGACCACATACCCAAATT
EsxF-PacI-FWD	ATATTTAATTAACAGAAAGGAGGATTTCAACTATCATGGGTGCCGACGACACGC
EsxF-W48A-FWD	TGTTGGGCGGGGCCCGCGGGGCGTCGGGC
EsxF-W48A-REV	GCCCGACGCCCCGCGGGCCCCGCCCAACA
EsxE-W38A-FWD	CATGTGACGGCCACGGGGGAG
EsxE-W38A-REV	CTCCCCCGTGGCCGTCACATG
EsxF-Y86A-FWD	GCCGGTGCGGGTGCCCAACACAACGAGACC
EsxF-Y86A-REV	GGTCTCGTTGTGTTGGGCACCCGCACCGGC
EsxE-87-90-Trunc-HindIII-REV	GCGGCCGAAGCTTTCACAAATTCGTGGCCA
EsxE-W89A-REV	AGTGCGGCCGAAGCTTTCACGAGGCCATACCCAAATTCGT
CpnT-pacI-FWD	ATATTTAATTAACAGAAAGGAGGATTTCAACTATCATGGCGCCGTTGGCGGTCGATCCC
IFT-hindIII-REV	GCAAGCTTTAACCCTTATAGTCCTTCCAAA
EsxF-HindIII-REV	ATATATAAGCTTTCAGCCACCGCCCACCTCACG
EsxE-PacI-FWD	ATATTTAATTAACAGAAAGGAGGATTTCAACTATCGTGGATCCGACCGTGTTG

Supplementary References

Bräuning B, Bertosin E, Praetorius F, Ihling C, Schatt A, Adler A, Richter K, Sinz A, Dietz H, Groll M. Structure and mechanism of the two-component α-helical pore-forming toxin YaxAB. Nat Commun. 2018 May 4;9(1):1806.

Danilchanka, O., Sun, J., Pavlenok, M., Maueroder, C., Speer, A., Siroy, A., Marrero, J., Trujillo, C., Mayhew, D.L., Doornbos, K.S., *et al.* (2014). An outer membrane channel protein of *Mycobacterium tuberculosis* with exotoxin activity. Proc Natl Acad Sci U S A *111*, 6750-6755.

Mitra, A., Speer, A., Lin, K., Ehrt, S., and Niederweis, M. (2017). PPE Surface Proteins Are Required for Heme Utilization by *Mycobacterium tuberculosis*. MBio *8*.

Sampson, S.L., Dascher, C.C., Sambandamurthy, V.K., Russell, R.G., Jacobs, W.R., Jr., Bloom, B.R., and Hondalus, M.K. (2004). Protection elicited by a double leucine and pantothenate auxotroph of *Mycobacterium tuberculosis* in guinea pigs. Infect Immun *72*, 3031-3037.

Schubert E, Vetter IR, Prumbaum D, Penczek PA, Raunser S. Membrane insertion of α-xenorhabdolysin in near-atomic detail. Elife. 2018 Jul 16;7:e38017.

Speer, A., Sun, J., Danilchanka, O., Meikle, V., Rowland, J.L., Walter, K., Buck, B.R., Pavlenok, M., Holscher, C., Ehrt, S., *et al.* (2015). Surface hydrolysis of sphingomyelin by the outer membrane protein Rv0888 supports replication of *Mycobacterium tuberculosis* in macrophages. Mol Microbiol *97*, 881-897.

Sun J, Siroy A, Lokareddy RK, et al. The tuberculosis necrotizing toxin kills macrophages by hydrolyzing NAD. *Nat Struct Mol Biol*. 2015;22(9):672-678.

Zhang K, Zhang H, Li S, Pintilie GD, Mou TC, Gao Y, Zhang Q, van den Bedem H, Schmid MF, Au SWN, Chiu W. Cryo-EM structures of *Helicobacter pylori* vacuolating cytotoxin A oligomeric assemblies at near-atomic resolution. Proc Natl Acad Sci U S A. 2019 Apr 2;116(14):6800-6805.
