# Supplementary material for: Pore-forming Esx proteins mediate toxin secretion by Mycobacterium tuberculosis
Source: Nat Commun. 2021 Jan 15;12:394. doi: 10.1038/s41467-020-20533-1 (PMC7810871; doi:10.1038/s41467-020-20533-1)
Supplement: Supplementary file 3 — Description of Additional Supplementary Files [file 41467_2020_20533_MOESM3_ESM.pdf]

Description of Additional Supplementary Files

File Name: Supplementary Data 1
Description: PDB files of all structural models
