# Supplementary material for: Pore-forming Esx proteins mediate toxin secretion by Mycobacterium tuberculosis
Source: Nat Commun. 2021 Jan 15;12:394. doi: 10.1038/s41467-020-20533-1 (PMC7810871; doi:10.1038/s41467-020-20533-1)
Supplement: Supplementary file 5 — Reporting Summary [file 41467_2020_20533_MOESM5_ESM.pdf]

nature research

Corresponding author(s): Michael Niederweis

Last updated by author(s): 11-19-20

Reporting Summary

Nature Research wishes to improve the reproducibility of the work that we publish. This form provides structure for consistency and transparency in reporting. For further information on Nature Research policies, see our Editorial Policies and the Editorial Policy Checklist.

Statistics

For all statistical analyses, confirm that the following items are present in the figure legend, table legend, main text, or Methods section.

n/a	Confirmed	
☐	☒	The exact sample size (n) for each experimental group/condition, given as a discrete number and unit of measurement
☐	☒	A statement on whether measurements were taken from distinct samples or whether the same sample was measured repeatedly
☐	☒	The statistical test(s) used AND whether they are one- or two-sided<br>*Only common tests should be described solely by name; describe more complex techniques in the Methods section.*
☐	☒	A description of all covariates tested
☐	☒	A description of any assumptions or corrections, such as tests of normality and adjustment for multiple comparisons
☐	☒	A full description of the statistical parameters including central tendency (e.g. means) or other basic estimates (e.g. regression coefficient) AND variation (e.g. standard deviation) or associated estimates of uncertainty (e.g. confidence intervals)
☐	☒	For null hypothesis testing, the test statistic (e.g. F, t, r) with confidence intervals, effect sizes, degrees of freedom and P value noted<br>*Give P values as exact values whenever suitable.*
☒	☐	For Bayesian analysis, information on the choice of priors and Markov chain Monte Carlo settings
☒	☐	For hierarchical and complex designs, identification of the appropriate level for tests and full reporting of outcomes
☒	☐	Estimates of effect sizes (e.g. Cohen's d, Pearson's r), indicating how they were calculated

Our web collection on statistics for biologists contains articles on many of the points above.

Software and code

Policy information about availability of computer code

Data collection	Lipid Bilayer Experiments - Data collected using CEC TestPoint on a custom-built electrophysiology apparatus.<br>Fluorescence microscopy was performed using a Zeiss Axiovert 200 with a 100x / 1.4 Plan apochromat coupled to a Zeiss Axiocam MRc camera.<br>Western blots were imaged using a Li-Cor Odyssey.<br>Flow cytometry was performed using a BD Accuri 6 cytometer.<br>Electron microscopy was performed using an FEI Tecnai F20 equipped with a K3 direct electron detector.
Data analysis	Lipid Bilayer Experiments - Data was analyzed using IGOR Pro 5.03<br>Fluorescence microscopy - Images were collected using Axiovision v4.5 software and analyzed further in ImageJ to create composites or perform maxima counting if necessary.<br>EM image analysis and reconstruction was performed using EMAN 2.2<br>Raw data for experiments was analyzed using Microsoft Excel when necessary or in Graphpad Prism v6.<br>All statistical analysis was performed using Graphpad Prism v6.

For manuscripts utilizing custom algorithms or software that are central to the research but not yet described in published literature, software must be made available to editors and reviewers. We strongly encourage code deposition in a community repository (e.g. GitHub). See the Nature Research guidelines for submitting code & software for further information.

Data

Policy information about availability of data

All manuscripts must include a data availability statement. This statement should provide the following information, where applicable:

- Accession codes, unique identifiers, or web links for publicly available datasets
- A list of figures that have associated raw data
- A description of any restrictions on data availability

Source data underlying figures is provided in an Excel file. The electron microscopy map has been deposited to EMDB (EMD-22727). Any other files or data are available upon request.

Field-specific reporting

Please select the one below that is the best fit for your research. If you are not sure, read the appropriate sections before making your selection.

☒ Life sciences ☐ Behavioural & social sciences ☐ Ecological, evolutionary & environmental sciences

For a reference copy of the document with all sections, see nature.com/documents/nr-reporting-summary-flat.pdf

Life sciences study design

All studies must disclose on these points even when the disclosure is negative.

Sample size	For fluorescence microscopy of infected macrophages we randomly selected at least 25 infected cells per infection experiment to score as TNT / EsxF / EsxE positive. The experiment was repeated 4 times for a total of at least 100 cells per infection.
Data exclusions	No data were excluded from analysis.
Replication	Experiments were performed at least 3 times to ensure reproducibility of findings unless otherwise indicated. For experiments which were performed twice, the results were already corroborated through an alternative experiment (IE: Flow cytometry, electron microscopy, lipid bilayer, etc) and were performed in order to visualize the results using multiple experimental approaches.<br>For surface staining experiments with EsxF and EsxE, a representative experiment is shown. The trend was reproduced over multiple experiments, but the number of bacteria per field of view (and consequently the EsxF or EsxE punctae / view) was different in each experiment thus preventing quantification of multiple experiments in a single histogram.<br>Lipid bilayer experiments were performed on at least 10 membranes using at least two separate preparations of protein. The number of membranes, insertions, etc. was determined based on our lab's numerous publications about channel-forming proteins.
Randomization	N/A
Blinding	The experimenters were not blinded during collection of the data. Our study does not involve animal or human subjects, thus blinding was not required. All experiments and data collection were performed by UT. EM data was validated by TD. All other data was validated by UT and MN.

Reporting for specific materials, systems and methods

We require information from authors about some types of materials, experimental systems and methods used in many studies. Here, indicate whether each material, system or method listed is relevant to your study. If you are not sure if a list item applies to your research, read the appropriate section before selecting a response.

Materials & experimental systems

n/a	Involved in the study	
☐	☒	Antibodies
☐	☒	Eukaryotic cell lines
☒	☐	Palaeontology and archaeology
☒	☐	Animals and other organisms
☒	☐	Human research participants
☒	☐	Clinical data
☒	☐	Dual use research of concern

Methods

n/a	Involved in the study	
☒	☐	ChIP-seq
☐	☒	Flow cytometry
☒	☐	MRI-based neuroimaging

Antibodies

Antibodies used	This information is described in supplementary table 3 with source, catalog number, and relevant publication.

Validation	The negative control strain (Mtb mc26206 - esxF-esxE-cpnT-ift deletion) was always included for western blots using TNT, esxF, or esxE, or IFT antibodies and were compared to the positive control (full operon complemented strain). Raw blots are available in the source data file. For GlpX, MctB, RNAP, and LpqH antibodies we used the molecular weights which have been determined through use of these markers in the literature, and in our laboratory's previous publications.

Eukaryotic cell lines

Policy information about cell lines

Cell line source(s)	THP-1 (ATCC TIB-202)
Authentication	THP-1 cell stocks were obtained from ATCC.
Mycoplasma contamination	THP-1 cells were negative for mycoplasma contamination (tested via PCR and DAPI staining).
Commonly misidentified lines (See ICLAC register)	*Name any commonly misidentified cell lines used in the study and provide a rationale for their use.*

Flow Cytometry

Plots

Confirm that:

- [x] The axis labels state the marker and fluorochrome used (e.g. CD4-FITC).
- [x] The axis scales are clearly visible. Include numbers along axes only for bottom left plot of group (a 'group' is an analysis of identical markers).
- [ ] All plots are contour plots with outliers or pseudocolor plots.
- [x] A numerical value for number of cells or percentage (with statistics) is provided.

Methodology

Sample preparation	Mycobacterium tuberculosis mc26206 was fixed with 4% PFA, blocked with 5% normal goat serum, stained with rabbit anti-TNT, and then stained with goat-anti-rabbit-FITC conjugate.
Instrument	BD Accuri 6
Software	FlowJo
Cell population abundance	No gating was performed since the sample contained only bacteria. A buffer control was performed. 1 million events were captured. The raw data is provided in the source data.
Gating strategy	No gating was performed since the sample contained only bacteria. A buffer control was performed. 1 million events were captured. The raw data is provided in the source data.

- [x] Tick this box to confirm that a figure exemplifying the gating strategy is provided in the Supplementary Information.
